# Integrating Conventional and Participatory Crop Improvement for Smallholder Agriculture Using the Seeds for Needs Approach: A Review

**DOI:** 10.3389/fpls.2020.559515

**Published:** 2020-09-15

**Authors:** Carlo Fadda, Dejene K. Mengistu, Yosef G. Kidane, Matteo Dell’Acqua, Mario Enrico Pè, Jacob Van Etten

**Affiliations:** ^1^ Biodiversity for Food and Agriculture, African Hub, Alliance of Bioversity International and CIAT, Nairobi, Kenya; ^2^ Biodiversity for Food and Agriculture, Alliance of Bioversity International and CIAT, Addis Ababa, Ethiopia; ^3^ Institute of Life Sciences, Scuola Superiore Sant’Anna, Pisa, Italy; ^4^ Dryland Crop and Horticultural Science, Mekelle University, Mekelle, Ethiopia; ^5^ Sirinka Agricultural Research Centre, Amhara Region Agricultural Research Institute (ARARI), Bahar Dar, Ethiopia; ^6^ Digital Inclusion, Alliance of Bioversity International and CIAT, Rome, Italy

**Keywords:** Seeds for Needs, genetic diversity, smallholder farmers, Ethiopia, participatory crop improvement, a high-tech crop improvement

## Abstract

In response to the climate change, it is essential to provide smallholder farmers with improved field crop genotypes that may increase the resilience of their farming system. This requires a fast turnover of varieties in a system capable of injecting significant amounts of genetic diversity into productive landscapes. Crop improvement is a pivotal strategy to cope with and adapt to climate change. Modern breeding may rely on the genomics revolution to speed up the development of new varieties with adaptive potential. However, centralized breeding may not adequately address smallholder farmers’ needs for more locally acclimatized varieties or groups of varieties. This, in turn, constrains adoption of new varieties that reduces the effectiveness of a resource-intensive breeding process, an issue that may be overcome with participatory, decentralized approaches. Whether high-tech centralized breeding or decentralized participatory approaches are better suited for smallholder farmers in the global South is hotly debated. Sidestepping any false dichotomies and ideological issues in these debates, this review provides a perspective on relevant advances in a breeding approach that combines the two approaches and uses genomics for trait mining from *ex situ* collections of genetic materials, participatory multilocation trials and crowdsourced citizen science. It argues that this new combination of high-tech centralized and participatory decentralized methods can provide a coherent and effective approach to breeding for climate adaptation and the present review advocates on a different way forward for the future research.

## Introduction

In many countries of the global South, there is a need to produce more food to adequately supply a fast-growing human population. However, in a business-as-usual scenario, crop yields are predicted to decline, soils will degrade even more, and new pests and diseases will appear (Ray et al., 2019). Humanity is in a “perfect storm”, as the food security challenge is compounded by the climate emergency (Godfray et al., 2010; Ripple et al., 2020). Crop improvement is expected to play a central role because of the need to develop new varieties better adapted to changing climates (Burke et al., 2009; Hickey et al., 2019). This should be achieved by taking into consideration that agriculture needs to deliver not only food and calories, but also nutrients to improve diets, reduce its greenhouse gas emissions, while maintaining or restoring soil fertility. It is therefore crucial to consider what kind of crop improvement strategy is best to effectively address 21^st^ Century agricultural challenges. Previous scientific literature distinguishes two apparently opposing crop improvement strategic approaches (Chiffoleau and Desclaux, 2006; Annicchiarico et al., 2019).

A first approach is “high-tech centralized breeding”: it relies on the technological advances that makes it possible to accelerate crop improvement. These includes harnessing genomics and phenotyping technologies, that allowed breeding to accelerate through the use of molecular markers and genomic selection (Abberton et al., 2016; Watson et al., 2018), and increasing genetic gains thanks to a higher selection intensity. These same methods can help in identifying specific traits for adaptation to climate change (Lopes et al., 2015). Rapid generation advance (RGA) makes it possible to have a larger number of selection cycles per year (up to 5.7 for wheat, 5.4 for barley, 3.8 for canola, and 4.5 for chickpea; Watson et al., 2018). These methods come together in the centralized development of improved crop varieties with high yield potential that later become globally distributed. At the moment, it takes over 10 years to develop a new variety of a cereal crop and make it available to the farmers in developing countries, before multiplying and delivering seed to farmers (Atlin et al., 2017).

A second approach is “decentralized participatory breeding”: it relies on the involvement of farmers to address the challenges that they face in marginal environments. Here, seed systems are largely informal and not connected to the seed businesses that intensively produce high-quality seeds of improved varieties. A study found that in a range of least developed countries (Democratic Republic of Congo, Haiti, Kenya, Malawi, South Sudan, and Zimbabwe), farmers get 90% of their seeds from informal sources (McGuire and Sperling, 2016). Much genetic diversity is still present on farms, the result of thousands of years of farmers’ conscious and unconscious selection (Jarvis et al., 2011). Vigouroux et al. (2011) found that farmer selection can support climate adaptation to shorten growing seasons. In such environments, centralized breeding efforts have had much more limited success: Ceccarelli (2015) provided evidence that decentralized approaches to breeding can increase its effectiveness and efficiency by enhancing adoption by farmers. On-farm selection eliminates the need for subsequent on-farm trials, accommodates farmer preferences, and allows capturing the interactions between genetic diversity, environment and management in target fields. Proponents of this second approach show that participatory methods can lead to increased rates of adoption (e.g., Sissoko et al., 2019). This approach relies on a broader use of farmers varieties, that have the following charactersitics: they are recognizable, distinct crop varieties; they have dynamic population character; they lack formal crop improvement; they are genetically heterogeneous; they are locally adapted; they are associated with local cultural, historic, or religious values; and they are associated with traditional farming systems (FAO, 2019).

Even though the two approaches are different from a conceptual and underlying philosophical point of views, there have been attempts to bring them together, recognizing the value of both (e.g., Dwivedi et al., 2017; Annichiarico et al., 2018). Among the fullest integrations, “Seeds for Needs” was developed as an integrated crop improvement initiative. By reviewing a range of studies published over the past few years and related to “Seeds for Needs”, the current study argues that the two breeding approaches can be reconciled into an innovative combined approach which can lead to higher breeding efficiency and accelerate adaptation to climate change. Finally, the current study will discuss how the proposed combined approach fits within the broader debate over future crop improvement.

## Reconciling High-Tech And Decentralized Breeding: The “Seeds for Needs” Approach

A key element of the “Seeds for Needs” approach is an emphasis on the intensive use of crop genetic diversity, combining high-tech, and decentralized methods. As breeding programs have matured, modern breeding relied on alleles selected from elite lines, making a limited use of alleles available in the wider genepool for adaptation to stress (Abberton et al., 2016). Many authors agree that traditional varieties are an important source of agronomic traits for adaptation to climate change and marginal growing conditions (e.g., Burke et al., 2009; Vasconcelos et al., 2013; Mengistu et al., 2019). Indeed, the genomic revolution allows breeders to cast a wider net to include more diversity into breeding programs and efficiently identifying adaptive traits and incorporating them into new varieties (Lopes et al., 2015). However, the resulting trait-based focus can quickly lose sight of the final goal: adaptation in target environments and farmer adoption of varieties. For example, Lopes et al. (2015) recognize the value of farmers’ varieties as sources for climate adaptation traits in wheat breeding and emphasizes the value of interdisciplinary collaboration to address multiple aspects, but do not discuss the importance of evaluation in target environments and evaluation against farmers’ needs. In this case, breeders’ selection was not sufficiently informed by varietal performance in target environments. On the other hand, farmer needs often comprise “tacit knowledge” that can only be accessed through participatory evaluation of varieties in the farmers’ field. This means that even when farmer varieties are used in breeding programs, the varieties that eventually reach farmers´ fields may still not necessarily address farmer needs if farmers are not involved in the process.

In 2010, Bioversity International^1^ started the “Seeds for Needs” approach as an attempt to leverage the genetic diversity in *ex situ* conservation facilities (genebanks) for climate adaptation in marginal environments, using durum wheat (*Triticum durum* Desf.) production areas in Ethiopia as a case study. The overarching aim of the approach was to cut short the crop improvement process by bringing genebank accessions directly to farmer fields for evaluation in target environments (Gotor et al., 2014). Since then, the Seeds for Needs approach has been implemented in a dozen countries in Africa, Asia, and Latin America (Kenya, Uganda, Ethiopia, Tanzania; Papua New guinea, India, Lao, Cambodia; Guatemala, Honduras, El Salvador, Nicaragua). Over time, multidisciplinary elements were added to this initiative that eventually grew into a broad, coherent approach that integrates genomics and participatory breeding methods pushing several innovations compared to the status quo.

As of today, the Ethiopian case study is the most developed within the Seeds for Needs portfolio. In that, the systematic sourcing of farmers’ varieties conserved *ex situ* proved to be a powerful tool for identifying and fast-tracking new varieties, which became useful to smallholder farmers’ communities. The study started with a molecular and phenotypic characterization of 373 farmer varieties conserved *ex situ* in the Ethiopian National Gene Bank managed by Ethiopian Biodiversity Institute (EBI) and 27 improved released varieties for cultivation in Ethiopia. The same panel was characterized for farmers’ preference using a Likert scale reporting appreciation of men and women wheat growers in relation to phenology, spike morphology and overall desirability of any wheat genotype. A total of 60 farmers (30 women and 30 men) were involved in the study. The main outcome of the study showed the following:

- Elevated genetic diversity was present in traditional wheat landraces cultivated in Ethiopia (Mengistu et al., 2016a; Mengistu et al., 2016b). This genetic diversity had not yet been fully exploited by breeders, as improved wheat varieties cultivated in Ethiopia were of Mediterranean origin with little or no introgression of traits found in varieties originated in Ethiopia. These results were later corroborated by other studies (Kabbaj et al., 2017) and may even support Ethiopia as a separate, secondary center of origin for the crop;
- In the studied environments, several farmer varieties were outperforming the modern varieties recommended by centralized breeding (Mengistu et al., 2019). Some farmer varieties performed over 5 t ha^−1^, which is double the average wheat productivity in Ethiopia. Moreover, landraces incorporated resistance traits against major wheat diseases, such as yellow and stem rust and septoria leaf blotch (*Zymoseptoria tritici* Desm.) (Kidane et al., 2017a);
- Local farmers could efficiently and consistently evaluate wheat varieties and provide measures significantly correlated with metric traits that are commonly measured by agronomists in breeding pipelines (Mancini et al., 2017);
- Local farmer evaluations were linked to quantitative traits, earliness, tillering capacity, and spike characteristics, linked to yield, with a genetic determination in wheat. An overall score was strongly correlated with biomas and yield components, thus with traits genetically determined. As such it was possible to trace the genetic basis of farmers’ preferred and scored traits which may or may not overlap with quantitative trait loci (QTL) responsible for agronomic traits of breeding relevance (Kidane et al., 2017b). It may, therefore, be deduced that QTLs derived from farmers scored traits may be incorporated into breeding pipelines to speed up the development of new varieties with genomic tools as per farmer needs.

These findings confirmed that useful traits may be available in farmer varieties held *ex situ*, that these traits are preferred by farmers, and that they can be made available for immediate use in pre-breeding with modern, genomics-based approaches. A further important step in the “Seeds for Needs” strategy in Ethiopia was the development of new durum wheat lines bringing together the genetic background of improved varieties from the international breeding gene pool with that of local farmer varieties. This approach was aimed at closing the gap resuling from the lack of consideration of useful traits from the Ethiopian genetic diversity. The approach taken here was to focus more closely on the local genepool rather than on globally representative sets of genetic materials, recognizing that local germplasm is closer to what farmers already use and need (Duvick, 1996) and may propel breeding for local adaptation.

Thus, a nested association mapping (NAM) population was created by crossing 50 Ethiopian farmer varieties with a single improved line with international pedigree, creating over 6,000 interconnected Recombinant Inbred Lines (RILs) sharing one parental haplotype. A preliminary characterization of 1,200 RILs from 12 families had shown that the more diverse and high yielding families are those bridging Ethiopian gene-pool with the international allele pool. The Ethiopian NAM, named EtNAM, has shown that it is possible to achieve significant steps in germplasm improvement by using alleles found in farmer varieties (Kidane et al., 2019).

The final element of the Seeds for Needs strategy used a citizen science or crowdsourcing approach to scale up the involvement of farmers in the evaluation of the varieties for local adaptation and to evaluate genetic materials directly in target environments. This approach proposed by Van Etten (2011) directly addresses some of the difficulties in more conventional participatory approaches, such as issues related with collective action to organize farmer groups, haphazard observation of common plots (Misiko, 2013), and limited validity and influence of decision-making by breeders (Sumberg et al., 2013). The approach involves the use of digital tools to scale farmer-participatory evaluation, individual, highly autonomous farmer participation rather than group-based approaches, and a widely distributed trial network (Van Etten et al., 2016; Steinke et al., 2017). A global synthesis of this approach involving 12,409 farmers from Ethiopia, India, and Nicaragua was reported by Van Etten et al. (2019). The study tested whether farmer citizen scientists could provide adequate, reliable information, which can be used to make recommendations for variety release that enhance farmers’ adaptation. Results showed that varieties with superior traits for tolerance to climate-induced stress could be identified with high effectiveness linking the farmer-generated data to climate data.

Increasing the number of stakeholders involved can directly link farmers to seed producers and allow extension services to deliver more customized support to farmers while empowering farmers on choosing what to sow in the next season. Citizen science, however, places additional responsibilities with farmers because they have to manage what could be defined as small experiments, including data collection. Often this happens by receiving very small amounts of seeds or varieties. In order to assess the potential for upscaling, it was important to understand farmers’ motivation to participate in a participatory action research project adding additional work to their schedule. Beza et al. (2017), showed that there are a number of factors that encouraged farmers from India, Honduras and Ethiopia to participate as citizen scientists, but they differ depending on the country. Being part of the research and contribute to it was the first motivation for Indian farmers, the second in Honduras, and the third in Ethiopia. In Ethiopia and Honduras, sharing the information with experts was the most important factor, clearly hoping to receive reciprocal advice. Indian and Ethiopian farmers were also keen to help as second most important motivational factor. Interestingly, the vast majority of farmers did not expect or demand any economic compensation.

Finally, the approach put strong emphasis on the strengthening of locally based seed systems, by supporting the establishment of community seeds banks to ensure adequate and timely availability of varieties by the community (Wasswa et al., 2015; McGuire and Sperling, 2016)

## Implications

This paper is rooted into a broader debate on how to reconcile high-tech centralized and decentralized participatory approaches, adding some new important elements. Dwivedi et al. (2017) and Ceccarelli (2015) already recognized the importance of using high-throughput phenotyping and molecular characterization during the pre-breeding stage of the breeding cycle and then promote participatory methodologies to achieve sustainable food production and more nutrient-dense varieties contributing to healthier diets, thus recognizing the need of combining different technological approaches during the breeding process. On the other hand, Atlin et al. (2017) argue that variety replacement should not only be accelerated using high-tech breeding but should also involve a much more assertive push of varieties to farms. The latter approach, however, could work only assuming a well-established breeding programme perfectly tuned into the needs and preferences of farmers and consumers, since a push approach *per se* does not allow an explicit articulation of varietal demand and does not address aspects related to local adaptation. Crop improvement is an important part of the seeds systems; thus, the way it is conducted has important repercussions on the entire food system (Mcguire and Sperling, 2013). Lammerts van Bueren et al. (2018) call for a system-based breeding approach which combines different breeding approaches to achieve ecological and societal resilience, arguing that breeding should contribute to food security, safety and quality, food and seed sovereignty, and social justice without jeopardizing long-term sustainability of the ecological context and should aim at enhancing agrobiodiversity, ecosystem services, and climate robustness. While it is beyond the scope of this review to address the complex issues of seed policy, seed soveregnity, and decommodification of seeds, the hitherto described “Seeds for Needs” approach is a step forward in designing a stepwise approach to “improve crop improvement” by combining different approaches with distinct features. From a purely selection point of view, this approach showed that participatory methods and the use of farmers’ acceptability improves the selection of suitable cultivars, inline with other studies (e.g., Annichiarico et al., 2018). From a broader perspective, it represents a decentralized, participatory, gender-sensitive modern breeding approach based on combining farmers’ knowledge with breeders’ observations, validated and fined-tuned through crowdsourcing.

The crowdsourcing approach allows to source data from multiple environments in a big data dimension. To achieve similar results with high-tech centralized breeding approaches, one would need to plan for larger multilocation trials at higher costs than those entailed by the participatory breeding approaches and crowdsourcing (also check Mangione et al., 2006 for a comparison of cost of paticipatory vs. non-participatory breeding programs). Additionally, high-tech centralized breeding methods lack the important and pervasive feedback from the farmers. Conversely, results from crowdsourcing approaches can be immediately actionable and effective, reducing the time needed for varieties to reach the farmers and providing a much faster turnover of varieties for different climatic conditions. This approach also identifies a portfolio of varieties that can be used to cope with climate unpredictability and for different types of abiotic stresses, pests or diseases. A portfolio approach at the community level, based on the use of mobile technology to keep monitoring the performance of varieties under variable climatic conditions, could link to a genetic database that would direct the appropriate crosses. In addition, it could also stimulate the creation of local seed businesses, possibly driven by new, greener, more socially responsible (Lammerts van Bueren et al., 2018).

If implemented in a feedback loop approach at the landscape or community level making use of information technology and big data, the reviewed approach has the potential to effectively innovate crop improvement. This approach would even support a formal technology-centered breeding program when appropriate. Since operational data would be based on local information, seeds could then be tested under the conditions for which they were developed, provided to farmers through crowdsourcing, released and integrated into the seed system through community seed banks and other local seed enteprises. Altogehter, this system would contribute to more integrated seed systems (both formal and informal), with more actors ensuring widers access to farmers’ preferred seeds (Mcguire and Sperling, 2013).

If superior traditional varieties can be found in *ex situ* collections (Mengistu et al., 2019), they could be released much quickly than in the situation in which a full breeding cycle is needed. Indeed, two varieties that were characterized by the Seeds for Needs initiative in 2011 and 2012 were released in 2017 after 5 years, i.e., 4 years faster than the average time required to release new varieties. Combining a system in which some superior traditional varieties are directly used by farmers while breeders can use them for further improvement, could inject more genetic diversity with adaptive traits in a shorter period time.

Finally, if the approach proposed here gains traction, there will be a strong push to support the characterization of the genetic resources conserved in national genebanks, most of which are still poorly, if at all, characterized, bridging a significant gap between conservation and utilization, it will promote crop improvement for locally important crops (Yu et al., 2016; Milner et al., 2019).

## Author Contributions

CF, DM, YK, MP, and MD’A developed the full protocol of Seed for Needs in Ethiopia. JE developed the tricot crowdsourced citizen science approach, and digital tools to implement the approach.

## Funding

Contract 81250391/A1424 - GIZ. Upscaling Access to Crowdsourced “Winner” Seed Varieties and Embedding Crowdsourcing in Ethiopian System as Delivery Mechanism for more Dynamic, Diverse and Market-Responsive Seed Portfolios. This review was possible with the contribution of CGIAR program on Water, Land and Ecosystem (WLE) and the CGIAR program on Climate Change, Agriculture and Food Security (CCAFS), which is carried out with support from the CGIAR Trust Fund and through bilateral funding agreements. For details please visit https://ccafs.cgiar.org/donors. The views expressed in this document cannot be taken to reflect the official opinions of these organizations.

## Conflict of Interest

The authors declare that the research was conducted in the absence of any commercial or financial relationships that could be construed as a potential conflict of interest.

## Footnotes

1 now the Alliance of Bioversity and CIAT
